# Analysis of Process Data of PISA 2012 Computer-Based Problem Solving: Application of the Modified Multilevel Mixture IRT Model

**DOI:** 10.3389/fpsyg.2018.01372

**Published:** 2018-08-03

**Authors:** Hongyun Liu, Yue Liu, Meijuan Li

**Affiliations:** ^1^Faculty of Psychology, Beijing Normal University, Beijing, China; ^2^Beijing Key Laboratory of Applied Experimental Psychology, Faculty of Psychology, National Demonstration Center for Experimental Psychology Education, Beijing Normal University, Beijing, China; ^3^Collaborative Innovation Center of Assessment Toward Basic Education Quality, Beijing Normal University, Beijing, China; ^4^Educational Supervision and Quality Assessment Research Center, Beijing Academy of Educational Sciences, Beijing, China

**Keywords:** computer-based problem solving, PISA2012, process data, the modified multilevel mixture IRT model, the process level, the student level

## Abstract

Computer-based assessments provide new insights into cognitive processes related to task completion that cannot be easily observed using paper-based instruments. In particular, such new insights may be revealed by time-tamped actions, which are recorded as computer log-files in the assessments. These actions, nested in individual level, are logically interconnected. This interdependency can be modeled straightforwardly in a multi-level framework. This study draws on process data recorded in one of complex problem-solving tasks (Traffic CP007Q02) in Program for International Student Assessment (PISA) 2012 and proposes a modified Multilevel Mixture IRT model (MMixIRT) to explore the problem-solving strategies. It was found that the model can not only explore whether the latent classes differ in their response strategies at the process level, but provide ability estimates at both the process level and the student level. The two level abilities are different across latent classes, and they are related to operational variables such as the number of resets or clicks. The proposed method may allow for better exploration of students' specific strategies for solving a problem, and the strengths and weaknesses of the strategies. Such findings may be further used to design targeted instructional interventions.

## Introduction

The problem-solving competence is defined as the capacity to engage in cognitive processing to understand and resolve problem situations where a solution is not immediately obvious. It includes the willingness to engage in these situations in order to achieve one's potential as a constructive and reflective citizen (OECD, 2014; Kurniati and Annizar, 2017). Problem solving can be conceptualized as a sequential process where the problem solver must understand the problem, devise a plan, carry out the plan, and monitor the progress in relation to the goal (Garofalo and Lester, 1985; OECD, 2013). These problem-solving skills are key to success in all pursuits, and they can be developed in school through curricular subjects. Therefore, it is no surprise that the problem-solving competency is increasingly becoming the focus of many testing programs worldwide.

Advances in technology have expanded opportunities for educational measurement. Computer-based assessments, such as simulation-, scenario-, and game-based assessments, constantly change item design, item delivery, and data collection (DiCerbo and Behrens, 2012; Mislevy et al., 2014). These assessments usually provide an interactive environment in which students can solve a problem through choosing among a set of available actions and taking one or more steps to complete a task. All student actions are automatically recorded in system logs as coded and time-stamped strings (Kerr et al., 2011). These strings can be used for instant feedback to students, or for diagnostic and scoring purposes at a later time (DiCerbo and Behrens, 2012). And they are called process data. For example, the problem solving assessment of PISA 2012, which is computer-based, used simulated real-life problem situations, such as a malfunctioning electronic device, to analyze students' reasoning skills, problem-solving ability, and problem-solving strategies. The computer-based assessment of problem solving not only ascertains whether students produce correct responses for their items, but also records a large amount of process data on answering these items. These data make it possible to understand students' strategies to the solution. So far, to evaluate students' higher order thinking, more and more large-scale assessments of problem solving become computer-based.

Recent research has focused on characterizing and scoring process data and using them to measure individual student's abilities. Characterizing process data can be conducted via a variety of approaches, including visualization, clustering, and classification (Romero and Ventura, 2010). DiCerbo et al. (2011) used diagraphs to visualize and analyze sequential process data from assessments. Bergner et al. (2014) used cluster analysis to classify similar behaving groups. Some other researchers used decision trees, neural networks, and Bayesian belief networks (BBNs) (Romero et al., 2008; Desmarais and Baker, 2012; Zhu et al., 2016), to classify the performance of problem solvers (Zoanetti, 2010) and to predict their success (Romero et al., 2013). Compared to characterizing process data, the research of scoring process data is very limited. Hao et al. (2015) introduced “the editing distance” to score students' behavior sequences based on the process data in a scenario-based task of the National Assessment of Educational Progress (NAEP). Meanwhile, these process data have been used in psychometric studies. Researchers analyzed students' sequential response process data to estimate their ability by combining Markov model and item response theory (IRT) (Shu et al., 2017). It is noteworthy that all these practices have examined process data that describe students' sequential actions to solve a problem.

All the actions, recorded as process level data, which are nested in individual level, are logically interconnected. This interdependency allows a straightforward modeling in a multi-level framework (Goldstein, 1987; Raudenbush and Bryk, 2002; Hox, 2010). This framework is similar to those used in longitudinal studies, yet with some differences. In longitudinal studies, measurements are typically consistent to show the development pattern of certain traits. For process data, however, actions are typically different within each individual. These successive actions are used to characterizing individuals' problem solving strategies.

It is common in computer-based assessments that a nested data structure exists. To appropriately analyze process data (e.g., time series actions) within a nested structure (e.g., process within individuals), the multi-level IRT model can be modified by allowing process data to be a function of the latent traits at both process and individual levels. It is noteworthy that in the modified model, the concept of “item” in IRT changed to each action in individuals' responses, which was scored based on certain rules.

With respect to the assessment of problem solving competency, the focus of this study is the ability estimate at the student level. We were not concerned with individual's ability reflected from each action at the process level, since the task needs to be completed by taking series actions. Even for individuals with high problem solving ability, the first few actions may not accurately reflect test takers' ability. As a result, more attention was put on the development of ability at the process level because it can reveal students' problem solving strategies. Mixture item response theory (MixIRT) models have been used in describing important effects in assessment, including the differential use of response strategies (Mislevy and Verhelst, 1990; Rost, 1990; Bolt et al., 2001). The value of MixIRT models lies in that they provide a way of detecting different latent groups which are formed by the dimensionality arising directly from the process data. These groups are substantively useful because they reflect how and why students responded the way they did.

In this study, we incorporated the multilevel structure into a mixture IRT model and used the modified multilevel mixture IRT (MMixIRT) model to detect and compare the latent groups in the data that have differential problem solving strategies. The advantage of this approach is the usage of latent groups. Although they are not immediately observable, these latent groups, which are defined by certain shared response patterns, can help explain process-level performance about how members of one latent group differ from another. The approach proposed in this study was used to estimate abilities both at process and student levels, and classify students into different latent groups according to their response strategies.

The goal of this study is to illustrate steps involved in applying the modified MMixIRT model in a computer-based problem solving assessment then to further present and interpret the results. Specifically, this article focuses on (a) describing and demonstrating the modified MMixIRT model using a task of PISA 2012 problem-solving process data; (b) interpreting the different action patterns; (c) analyzing the correlation between characteristics of different strategies and task performance, as well as some other operational variables such as the number of resets or clicks. All the following analysis was based on one sample data set.

## Measurement material and dataset

### Problem solving item and log data file

**Figure 1:**
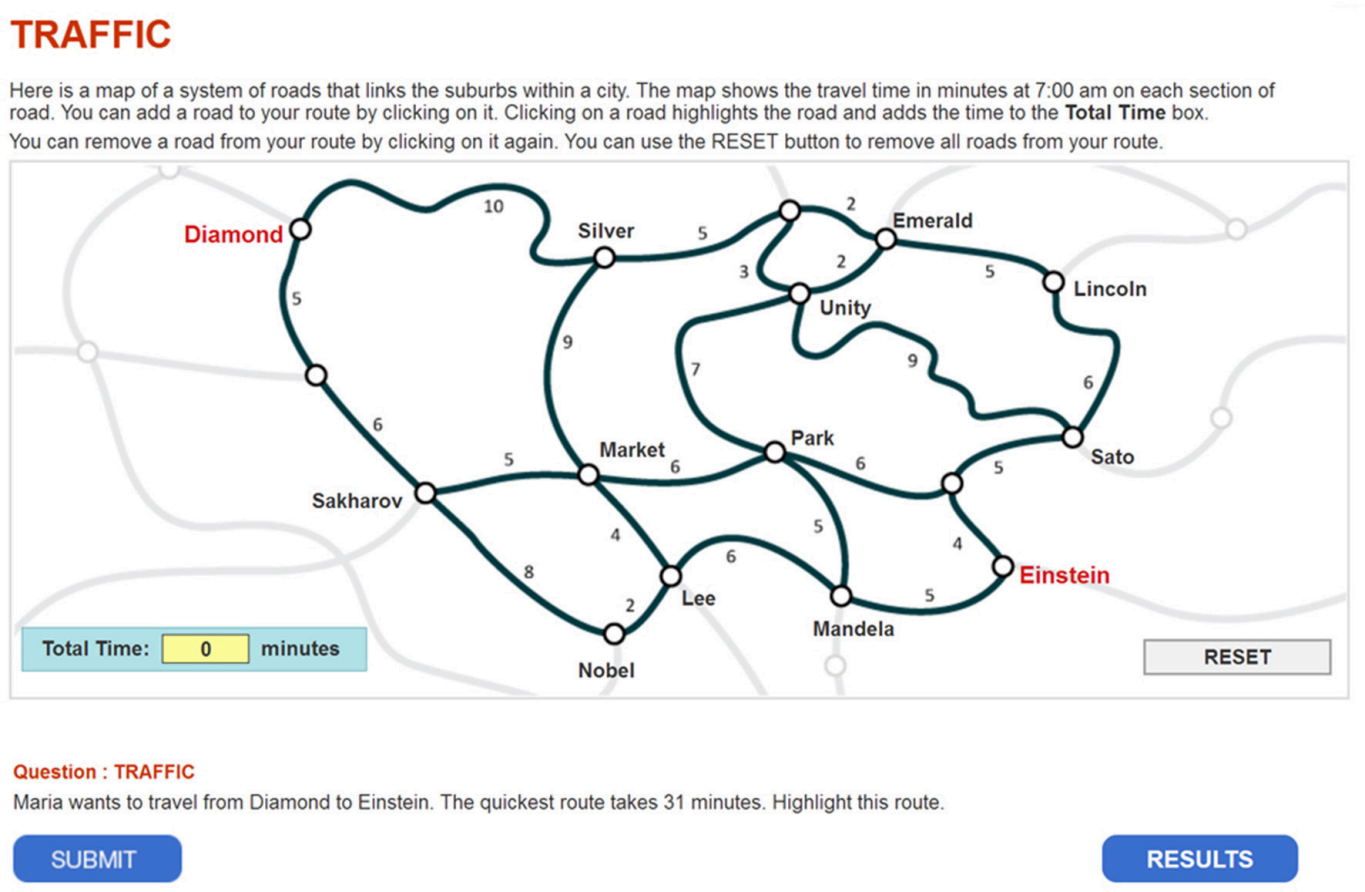
Traffic.

This study illustrates the use of the modified MMixIRT model in analyzing process data through one of the problem-solving tasks in PISA 2012 (Traffic CP007Q02). The task is shown in Figure 1. In this task, students were given a map and the travel time on each route, and then they were asked to find the quickest route from Diamond to Einsten, which takes 31 min.

The data are from the task's log file (CBA_cp007q02_logs12_SPSS.SAV, data source: http://www.oecd.org/pisa/data/) (an example of log data file is shown in Appendix 1). The data file contains four variables associated with the process. The “event” variable refers to the type of event, which may be either system generated (start item, end item) or student generated (e.g., ACER_EVENT, Click, Dblclick). The “time” variable is the event time for this item, given in seconds since the beginning of the assessment, with all click and double-click events included. The “event_value” variable is recorded in two rows, as a click event involves selecting or de-selecting a route of the map. For example, in the eleventh row where the state of the entire map is given, 1 in the sequence means that the route was selected, and 0 means that it was not; the twelfth row records an event involving highlighting, or un-highlighting. A route of the map represents the same click event, and it is in the form “hit_segment name” (The notes on log file data can be downloaded from http://www.oecd.org/pisa/data/). All the “click” and “double-click” events represent that a student performs a click action that is not related to select a route. Table 1 shows the label, the route and the correct state of the entire selected routes.

**Table 1:** The routes of the map.

**Label**	**Route**	**Included or not in the correct routes**
P1	Diamond-Nowhere	1
P2	Diamond-Silver	0
P3	Emerald-Lincoln	0
P4	Emerald-Unity	0
P5	Lee-Mandela	1
P6	Lincoln-Sato	0
P7	Mandela-Einstein	1
P8	Market-Lee	1
P9	Market-Park	0
P10	Nobel-Lee	0
P11	Nowhere-Einstein	0
P12	Nowhere-Emerald	0
P13	Nowhere-Sakharov	1
P14	Nowhere-Unity	0
P15	Park-Mandela	0
P16	Park-nowhere	0
P17	Sakharov-Market	1
P18	Sakharov-Nobel	0
P19	Sato-nowhere	0
P20	Silver-Market	0
P21	Silver-nowhere	0
P22	Unity-Park	0
P23	Unity-Sato	0

*1, Yes; 0, No*.

### Sample

The study sample was drawn from PISA 2012 released dataset, consisting of a total of 413 students from 157 American schools who participated in the traffic problem-solving assessment (47.2% as females). The average age of students was 15.80 years (*SD* = 0.29 years), ranging from 15.33 to 16.33 years.

For the traffic item response, the total effective sample size under analysis was 406, after excluding seven incomplete responses. For the log file of the process record, there were 15,897 records in the final data file, and the average record number for each student was 39 (*SD* = 33), ranging from 1 to 183. The average response time was 672.64 s (*SD* = 518.85 s), ranging from 58.30 to 1995.20 s.

## The modified mmixirt model for process data

### Process-level data coding

In this task log file, “ACER_EVENT” is associated with “click.” However, in this study we only collected the information of ACER_EVENT and deleted the redundant click data. Then, we split and rearranged the data by routes, making each row represent a step in the process of individual students, and each column represent a route (0 for de-selecting, and 1 for selecting). Table 2 shows part of the reorganized data file, indicating how individual student selected each route in each step. For example, the first line represents that student 00017 selected P2 in his/her first step.

**Table 2:** Example of the reorganized data file.

**StIDStd**	**Time**	**Event_number**	**Event_value**	**P1**	**P2**	**P3**	**P4**	**P5**	**P6**	**P7**	**P8**	**…**	**P21**	**P22**	**P23**
00017	837.6000	2.00	'01000000000000000000000	0	1	0	0	0	0	0	0	**…**	0	0	0
00017	839.8000	4.00	'11000000000000000000000	1	1	0	0	0	0	0	0	**…**	0	0	0
00017	841.1000	7.00	'11000000000010000000000	1	1	0	0	0	0	0	0	**…**	0	0	0
00017	841.7000	9.00	'11000000000010000100000	1	1	0	0	0	0	0	0	**…**	0	0	0
00017	842.7000	11.00	'11000000010010000100000	1	1	0	0	0	0	0	0	**…**	0	0	0
00017	844.8000	13.00	'11000000010010000101000	1	1	0	0	0	0	0	0	**…**	0	0	0
00017	846.4000	15.00	'11000000010000000101000	1	1	0	0	0	0	0	0	**…**	0	0	0
00017	847.4000	17.00	'01000000010000000101000	0	1	0	0	0	0	0	0	**…**	0	0	0
00017	848.4000	19.00	'01000000010000000001000	0	1	0	0	0	0	0	0	**…**	0	0	0
00017	850.6000	21.00	'01000000000000000001000	0	1	0	0	0	0	0	0	**…**	0	0	0
00017	851.6000	23.00	'01000000010000000001000	0	1	0	0	0	0	0	0	**…**	0	0	0
00017	852.5000	25.00	'01000000000000000001000	0	1	0	0	0	0	0	0	**…**	0	0	0
00017	853.4000	27.00	'01000000100000000001000	0	1	0	0	0	0	0	0	**…**	0	0	0
00017	853.7000	29.00	'01000000100000010001000	0	1	0	0	0	0	0	0	**…**	0	0	0

Process data were first recoded for the analysis purpose. Twenty-three variables were created to represent a total number of available routes that can possibly be selected (similar to 23 items). The right way for solving this problem is to select the following six routes: Diamond–Nowhere–Sakharov–Market–Lee–Mandela–Einstein (i.e., P1, P5, P7, P8, P13, and P17). For the correct routes, the scored response was 1 if one was selected, and 0 otherwise; for the incorrect routes, the scored response was 0 if one was selected, and 1 otherwise. Each row in the data file represents an effective step (or action) a student took during the process. In each step, when a route was selected or not, the response for this route was recoded accordingly. When a student finished an item, all the steps during the process were recorded. Therefore, for the completed data set, the responses of the 23 variables were obtained and the steps were nested within students.

### The modified MMixIRT model specification

The MMixIRT model has mixtures of latent classes at the process level or at both process and student levels. It assumes that possible heterogeneity exists in response patterns at the process level and therefore are not to be ignored (Mislevy and Verhelst, 1990; Rost, 1990). Latent classes can capture the interactions among the responses at the process level (Vermunt, 2003). It is interesting to note that if no process-level latent classes exist, there are no student-level latent classes, either. The reason lies in that student-level units are clustered based on the likelihood of the processes belonging to one of the latent classes. For this particular consideration, the main focus in this study is to explore how to classify the process-level data, and the modified MMixIRT model only focus on latent classes at the process level.

The MMixIRT model accounts for the heterogeneity by incorporating categorical or continuous latent variables at different levels. Because mixture models have categorical latent variables and item response models have continuous latent variables, latent variables at each level may be categorical or continuous. In this study, the modified MMixIRT includes both categorical (latent class estimates) and continuous latent variables at the process level and only continuous (ability estimates) latent variables at the student level.

The modified MMixIRT model for process-level data is specified as follows:

Process-Level

(1) $$\begin{array}{r} \text{P}\left(y_{jki}=1|\theta_{jkg},C_{jk}=g\right)=\frac{\text{exp}\left(\alpha_{ig.W}\theta_{jkg}-\beta_{ig}\right)}{1+\text{exp}\left(\alpha_{ig.W}\theta_{jkg}-\beta_{ig}\right)} \end{array}$$

$$\begin{array}{r} P\left(y_{jk1}=\omega_{1},y_{jk2}=\omega_{2},\cdots \;,y_{jkI}=\omega_{I}\right)=\\ \sum_{g=1}^{G}\gamma_{jkg}\prod_{i=1}^{I}{P\left(y_{jki}=1|\theta_{jkg},C_{jk}=g\right)}^{\omega_{i}} \end{array}$$

(2) $$\begin{array}{r} {\left(1-P\left(y_{jki}=1|\theta_{jkg},C_{jk}=g\right)\right)}^{\left(1-\omega_{i}\right)} \end{array}$$

Student-Level

(3) $$\begin{array}{r} \text{P}\left(y_{ki}=1|\theta_{k}\right)=\frac{\text{exp}\left(\alpha_{i.B}\theta_{k}-\beta_{i}\right)}{1+\text{exp}\left(\alpha_{i.B}\theta_{k}-\beta_{i}\right)} \end{array}$$

For the process level, in Equation (1), ***i*** is an index for *i*th route (*i* = 1, …, *I*), ***k*** is an index for a student (*k* = 1,…, *K*), ***j*** is an index for the *j*th valid step of a student during the response process (*j* = 1, …, *J*_*k*_),(*J* is the total steps of the *k*th student) and *g* indexes the latent classes (*C*_*jk*_ = 1, …, *g*…*G*, where *G* is the number of latent classes), ***C***_*jk*_ is a categorical latent variable at the process level for the *j*th valid step of student *k*, which captures the heterogeneity of the selections of routes in each step. *P*(*y*_*jki*_ = 1|θ_*jkg*_, *C*_*jk*_ = *g*) is the probability of selecting an route *i* in the *j*th step of student *k*, which is predicted by the two-parameter logistic (2PL) model, and α_*ig*.*W*_ is the discrimination parameter of process-level in class *g, W* means within-level, β_*ig*_ is the location parameter in class *g*, and θ_*jkg*_ is the latent ability of examinee ***k*** for a specific step ***j*** during the process of selecting the route, which is called the process ability in this study (θ_*jkg*_ ~***N(***μ_*jkg*_**,** $\sigma_{jkg}^{2}$***)***). The process abilities across different latent classes are constrained to follow a normal distribution ***(***θ_*jk*_ ~***N(0, 1))*.** In Equation (2), *P*(*y*_*jk*1_ = ω_1_, *y*_*jk*2_ = ω_2_, ⋯ , *y*_*jkI*_ = ω_*I*_) is the joint probability of the actions in the *j*^th^ step of student *k*. ω_*i*_ denotes either selected or not selected for *i*th route. For the correct routes, 1 represents that the route was selected, and 0 otherwise; for the incorrect routes, 0 represents that the route was selected, and 1 otherwise. γ_*jkg*_ is the proportion of the *j*th step in each latent class and $\overset{G}{\underset{g=1}{\sum }}\gamma_{jkg}=1$. As can be seen from the Equation (2), the probability of the actions (*y*_*jki*_) are assumed to be independent from each other given class membership, which is known as the local independence assumption for mixture models.

For the student level, in Equation (3), α_*i*.*B*_ is the item discrimination parameter where ***B*** represents between-level. β_*i*_ is the item location parameter which is correlated with the responses of the final step of the item. θ_*k*_ is the ability estimate at the student level based on the final step of the process, which also represents the problem-solving ability of student ***k*** in this study ***(***θ_*k*_ ~***N(0*,** ***1))***.

**Figure 2:**
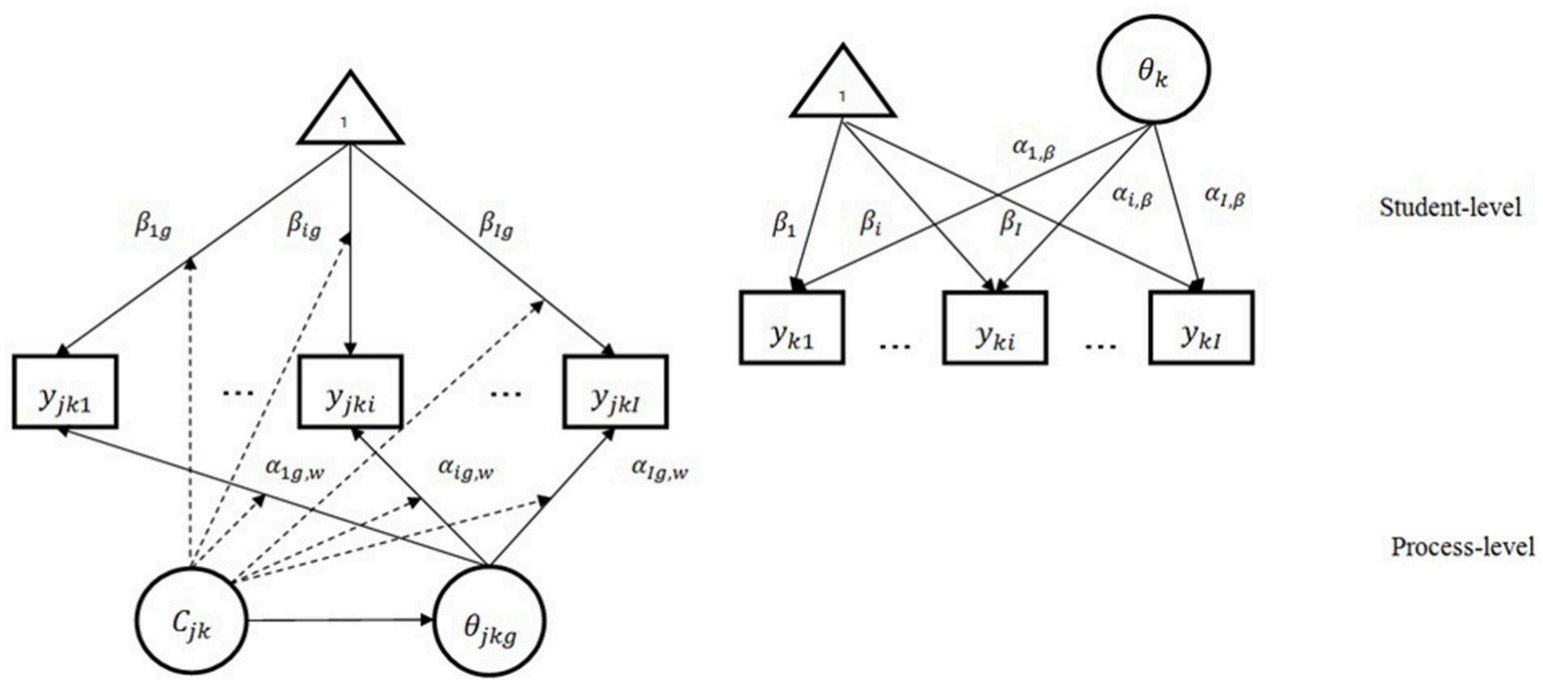
The modified MMixIRT model for process data.

Figure 2 demonstrates a modified two-level mixture item response model with within-level latent classes. The squares in the figure represent item responses, the ellipses represent latent variables, and 1 inside the triangle represents a vector of 1 s. As is shown in the figure, the response for each route of the jth step [***y***_*jk*1_**,…,** ***y***_*jki*_**,…,** ***y***_*jkI*_] is explained by both categorical and continuous latent variables (***C***_*jk*_ and θ_*jkg*_, respectively) at the process level; and the final response of students for each route [***y***_*k*1_**,…,** ***y***_*ki*_**,…,** ***y***_*kI*_] is explained by a continuous latent variable (θ_*k*_) at the student level. The arrows from the continuous latent variables to the item (route) represent item (route) discrimination parameters (α_*ig, W*_ at the process level and α_*i, B*_ at the student level), and the arrows from the triangle to the item responses represent item location parameters at both levels. The dotted arrows from the categorical latent variable to the other arrows indicate that all item parameters are class-specific.

It should be noted that the MMixIRT model is different from the traditional two-level mixture item response model in the definition of the latent variables at the between-level. In the standard MMixIRT model, the between-level latent variables are generally obtained from the measurement results made by within-level response variables [***y***_*jk*1_**,…,** ***y***_*jki*_**,…,** ***y***_*jkI*_] on between-level latent variables (Lee et al., 2017). In this study, the process-level data mainly reflect the strategies for problem solving, while the responses at the last step represent students' final answers on this task. Therefore, students' final responses are used to estimate their problem-solving abilities (latent variable at the between-level, i.e., ability of the student level) in the modified MMixIRT model.

Mplus Software (Muthén and Muthén, 1998-2015) was used to estimate the parameters of the modified MMixIRT model, as specified above. In addition, the detailed syntax are presented in Appendix 5.

## Results

### Results of descriptive statistics

Table 3 shows the proportion of each route selected by the students in the correct group and in the wrong group, respectively. The correct group consists of students who selected the right routes, and the wrong group refers to students who failed to do so. There are a total of 476 students, with 377 in the correct group and 99 in the wrong group. The results show that most of the students in the correct group selected the right routes, while a large number of students in the wrong group selected the wrong routes. To further explore the differences of the proportion of students selecting the wrong routes in the two groups, χ^2^-tests were conducted. No significant differences were found between the correct group and the wrong group in terms of the proportion of students who clicked four wrong routes, including P4 [χ^2^_(1)_ = 0.370, *P* > 0.05], P9 [χ^2^_(1)_ = 3.199, *P* > 0.05], P10 [χ^2^_(1)_ = 3.636, *P* > 0.05], and P15 [χ^2^_(1)_ = 2.282, *P* > 0.05]. This further suggests that it was difficult for the correct group to avoid these routes during their response process, and even quite a number of students in the correct group experienced trial and error before eventually solving the problem.

**Table 3:** The proportion of route selection.

**Route**	**Selected proportion**	
	**Wrong group**	**Correct group**
**P1**	**40.023**	**69.504**
P2	38.158	19.872
P3	3.290	1.688
P4	0.635	0.815
**P5**	**16.055**	**25.148**
P6	2.481	1.287
**P7**	**15.699**	**22.260**
**P8**	**4.340**	**21.953**
P9	25.379	23.435
P10	12.586	12.007
P11	16.559	10.819
P12	4.304	2.601
**P13**	**36.846**	**64.109**
P14	8.404	3.622
P15	5.182	6.886
P16	19.122	12.771
**P17**	**16.653**	**43.530**
P18	17.629	13.157
P19	4.884	1.923
P20	17.579	10.732
P21	15.369	7.211
P22	5.531	1.759
P23	4.296	1.377

*The right routes are printed in bold*.

### Results of the modified MMixIRT model

#### Model selection

The determination of the number of latent classes has been discussed in many studies (Tofighi and Enders, 2008; Li et al., 2009; Peugh and Fan, 2012). Several statistics of the mixture IRT models are often computed to compare relative fits of these models. Akaike's (1974) information criterion (AIC) incorporates a kind of penalty function for over-parameterization on model complexity. A criticism of AIC has been that it is not asymptotically consistent because the sample size is not directly involved in its calculation (Janssen and De Boeck, 1999; Forster, 2004). Schwarz (1978) proposed BIC as another information-based index, which attains asymptotic consistency by penalizing over-parameterization by using a logarithmic function of the sample size. For the sample size in BIC, the number of persons is used in multilevel model (Hamaker Ellen et al., 2011) and in multilevel item response model (Cohen and Cho, 2016). Most studies suggested the BIC value as the best choice because it was a sample-based index that also penalized the sophisticated model. However, Tofighi and Enders (2008) indicated in their simulation study that a sample size-adjusted BIC (aBIC) was an even better index. Smaller AIC, BIC, and aBIC values indicate a better model fit for mixture IRT models. Besides, entropy value has been used to measure how well a mixture model separates the classes; an entropy value close to 1 indicates good classification certainty (Asparouhov and Muthén, 2014).

The model selection results for the modified MMixIRT models are given in Table 4. The model fit indicates that *LL, AIC, BIC*, and *aBIC* decreased consistently as the class number increased to eight classes, and the nine-class model did not converge. As noted above, the best fit for *AIC, BIC*, and *aBIC* was determined or dictated by the smallest value in the ordered set of models from the least to the most complex. As suggested by Rosato and Baer (2012), selecting a robust latent class model is a balance between the statistical result of the model fit and the substantive meaning of the model. The model that fits best and yields meaningful classes should be retained. In this study the proportions of latent classes were examined to ensure the empirical significance, and the interpretability of each class was considered accordingly. For the 6-class model, the proportion of each class was 18.1, 30.7, 18.1, 20.1, 7.2, and 5.9%. And for the 7-class model, the proportion was 19.9, 13.4, 6.0, 12.3, 13.5, 27.4, and 7.5%. Compared to the 6-class model, in the 7-class model, the extra class of the steps was similar to class 2 of the 6-class model, while mixing class 4 at the same time. This makes the 7-class model hard to interpret. For the 8-class model, the proportion of one of the classes was too small (only 2.7%). Taking into account both the model fit index and the interpretability of each class, the 6-class model was retained in this study.

**Table 4:** Model comparison and selection.

**No of class**	**No of Free parameters**	***LL Value***	***Akaike (AIC)***	***Bayesian (BIC)***	***Sample-Size Adjusted BIC***	***Entropy***
1	46	−112745.581	225583.161	225936.108	225789.923	
2	95	−99334.232	198858.463	199587.375	199285.472	0.957
3	144	−92723.338	185734.676	186839.552	186381.931	0.860
4	193	−89375.035	179134.070	180607.239	179997.077	0.920
5	242	−87186.912	174857.823	176714.629	175945.571	0.936
6	291	−85974.117	172530.234	174763.005	173838.228	0.908
7	340	−84864.882	170409.764	173018.500	171938.004	0.904
8	389	−83821.533	168421.066	171405.766	170169.552	0.893

#### Description of class characteristics

The most likely latent class membership are displayed in Table 5. In this matrix, steps from each class have an average probability of being in each class. Large probabilities are expected on the diagonal. The numbers on diagonal are greater than 0.9. It can be concluded from the results that the modified MMixIRT model can classify students properly based on process data.

**Table 5:** Most likely latent class membership of each class.

	**Most likely latent class membership**					
	**Class 1**	**Class 2**	**Class 3**	**Class 4**	**Class 5**	**Class 6**
Class 1	0.945	0.000	0.006	0.033	0.004	0.012
Class 2	0.001	0.936	0.002	0.033	0.013	0.015
Class 3	0.002	0.020	0.949	0.011	0.017	0.001
Class 4	0.029	0.004	0.007	0.949	0.002	0.010
Class 5	0.002	0.007	0.018	0.002	0.969	0.002
Class 6	0.016	0.014	0.001	0.025	0.002	0.942

**Figure 3:**
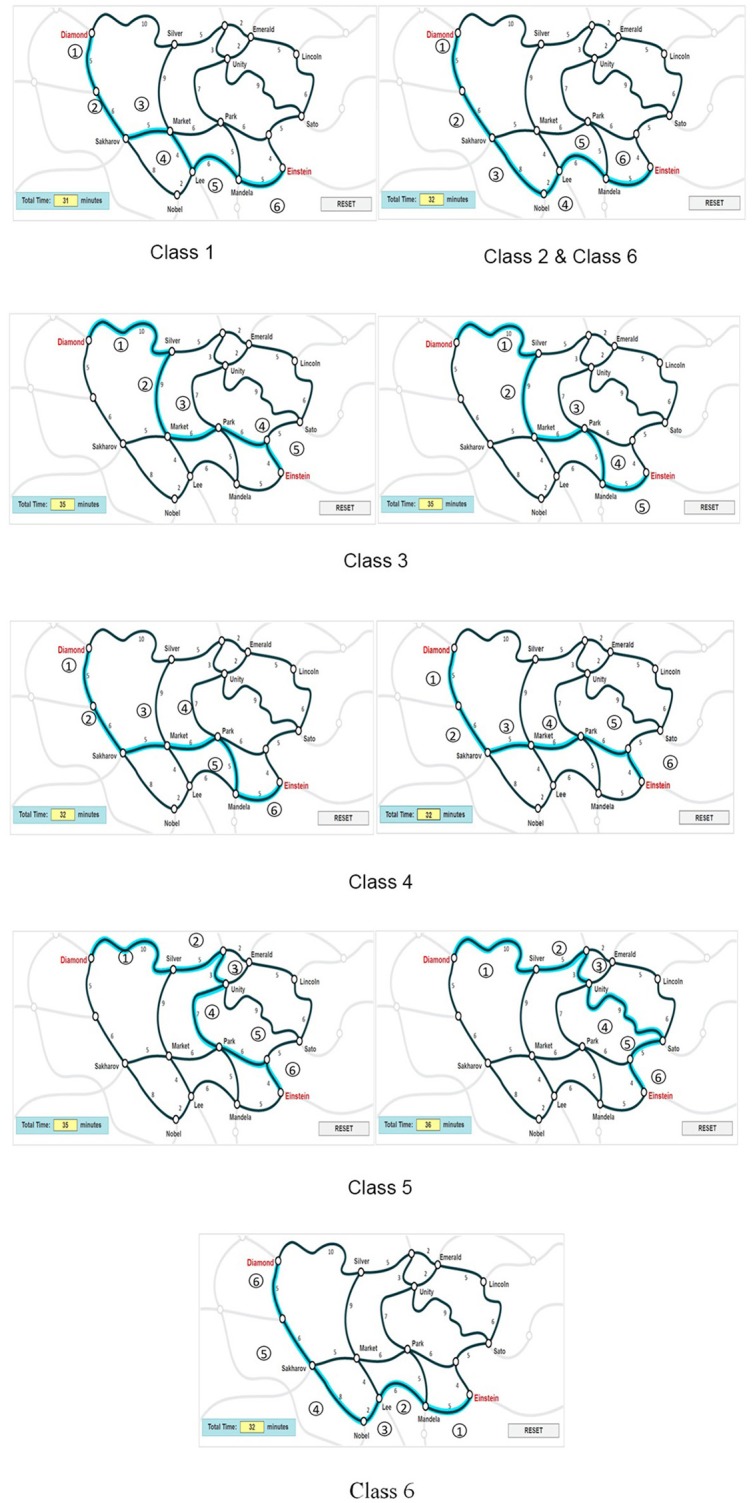
Route selection strategy by class.

Figure 3 presents the characteristics of route selection for each class based on the 6-class mixture IRT model, with ➀, ➁, ➂.…indicating the order of the routes. Based on the results of the modified MMixIRT model, the number of clicks of the 23 routes (P1–P23) in each class is listed in Appendix 2. The characteristics of route selection can be obtained pursuant to routes that get more clicks than others in each class, as well as the relations among routes shown in Figure 1. For example, P17, P13, P1, P8, P5, P16, and P7 in Class 1 were clicked more than other routes; however, Figure 1 shows that there is no obvious relationship between P16 and other routes. Therefore, the characteristic of Class 1 was defined as P1-P13-P17-P8-P5-P7 and P16 was removed. These routes were sequenced by the number of clicks they got, with the most clicked routes taking the lead. As indicated in Figure 3, different latent classes have typical characteristics depending on the similarity of the correct answers. For example, the route selection strategy of Class 1 best approximated the ideal route required by the item. Based on their last click, almost all the students in Class 1 gave the correct answer. Therefore, Class 1 could be regarded as the correct answer class, while the rest classes took different wrong routes.

The numbers in circles (➀, ➁, ➂….) indicate the order of the routes.

As is illustrated in Table 6, different classes demonstrated different means of process-level ability. It is obvious that the mean process ability in Class 1 is the highest (0.493), followed by Class 6, Class 2, Class 4, yet Class 5 and Class 3 with the lowest process-level ability. A closer check of these classes in Figure 3 indicates that the selected routes of Class 5 and Class 3 were incredibly far away from the correct one, and they took far more than 31 min. Therefore, it is no surprise that the mean process-level ability estimates of these two classes were the lowest and were both negative (−1.438 and −0.935, respectively). In addition, as can be seen in the number of students, almost all the students in Class 1 provided the right answer, demonstrating that different latent classes had different probabilities of the correct answer. In summary, the process-level ability is different across latent classes, which is related to different strategies of students' route selection or cognitive process.

**Table 6:** Means and standard deviations of process level abilities.

	**Latent class size for process-level**		**No of Students**		**Process-level ability**	
	**Count**	**Proportion**	**Right**	**Wrong**	**Mean**	***SD***
Class 1	2875	18.1	307	3	0.493	0.678
Class 2	4867	30.7	0	41	0.323	0.903
Class 3	2867	18.1	0	14	−0.935	0.386
Class 4	3192	20.1	0	26	0.292	0.556
Class 5	1138	7.2	0	12	−1.438	0.404
Class 6	940	5.9	0	3	0.424	0.698
Total	15879	100	307	99	0.000	0.934

*In the column of no of Students, the last step of the process within each student is classified into one of the six latent classes. Then, the numbers of students who gave the correct or wrong answer are summarized based on the latent classes*.

#### The sequence of latent classes at the process level

Based on the results of the modified MMixIRT model, the characteristics of the strategy shifts between step-specific classes were explored and summarized. To capture the characteristics of students' strategy shifts during the response, it is necessary to identify the typical route selection strategy of each class in the first place. In this study, if a student applied the strategy of a certain class three or more times consecutively, it was considered that the student had employed the strategy of this class at the process level. Three times was chosen as the rule of thumb because it demonstrated enough stability to classify a solution behavior. Then the strategy shifts of each student during their clicking procedure could be obtained in orders. The typical route selection strategy of different classes and the class shifts of students in the correct group are presented in Appendixes 3, 4, respectively. The results in Appendix 4 provide useful and specific information about the strategy shifts used by students over time. For example, in the correct group, 58 students shifted from one class to another, including 22 from Class 2 to Class 1, 3 from Class 3 to Class 1, 30 from Class 4 to Class 0, and 3 from Class 6 to Class 1. It is noteworthy that when students did not apply any strategies for more than three times consecutively, it was regarded as class 0 in this study.

### The relationship of the two level ability estimates and operational variables

To validate whether students with different patterns of actions will have different process-level ability, the descriptive statistics were conducted of operational variables such as the number of route clicks and resets and their correlation with the mean ability estimate of process-level ability (See Table 7 for details). To further explore the differences of click actions between the correct group and the wrong group, several *T*-tests were conducted. The results indicate that students in the correct group did significantly fewer resets than their counterparts in the wrong group [*t*_(404)_ = 2.310, *P* < 0.05]. No significant differences were detected of the number of routes clicked or the response time between the correct group and the wrong group [*t*_(404)_ = 1.656, *P* = 0.099; *t*_(404)_ = −0.199, *P* = 0.843]. The results in Table 7 suggest two things. Firstly, positive correlation existed between the estimate of student-level ability and that of process-level ability. This means that the process-level ability estimate provides consistency and auxiliary diagnostic information about the process. The students with higher process-level ability had higher ability estimates of student level. Secondly, for the process-level ability, a significant negative correlation existed between the mean process-level ability estimate and variables such as the valid number of route clicks and the number of resets for students in the correct group. It is concluded that in the correct group, the less frequently a student clicks the routes and resets the whole process, the higher process-level ability he or she is likely to obtain. For students in the wrong group, however, no significant correlations were observed between the mean ability estimate and the variables discussed above. Instead, a significant negative correlation was found between the mean process-level ability estimate and the absolute time of difference from 31 min. For these students, their process-level ability decreased as the time cost by the wrong routes increased. Third, the mean process-level ability estimate for the correct group was 0.310, in contrast to −0.175 for the wrong group, which reveals a significant difference between the two groups [*t*_(404)_ = 8.959, *P* < 0.001]. In terms of student-level ability, the estimate for the correct group was significantly higher than for the wrong group [*t*_(404)_ = 112.83, *P* < 0.001].

**Table 7:** Correlation between ability estimates and operational variables in process.

**Item response result**	**Click action variable**	**Mean ability of process level**	**Ability of student level**	**Mean**	***SD***
Correct (*N* = 307)	No of Route Clicks	−0.657^**^	/	79.760	63.874
	No of Resets	−0.467^**^	/	0.919	1.737
	Absolute Time of Difference from 31	**/**	/	0.000	0.000
	Response Time	0.048	/	675.540	525.710
	Mean Ability of Process Level	/	/	0.310	0.447
	Ability of Student Level	**/**	/	1.371	0.000
Wrong (*N* = 99)	No of Route Clicks	−0.050	0.142	93.030	84.138
	No of Resets	−0.124	0.098	1.394	1.910
	Absolute Time of Difference from 31	−0.248^*^	−0.179	5.210	10.869
	Response Time	−0.087	0.022	663.620	499.466
	Mean Ability of Process Level	/	0.597^**^	−0.175	0.530
	Ability of Student Level	0.597^**^	/	−0.432	0.281
Total (*N* = 406)	No of Route Clicks	−0.439^**^	−0.066	83.000	69.484
	No of Resets	−0.378^**^	−0.103^*^	1.035	1.790
	Absolute Value of Difference from 31	−0.269^**^	−0.407^***^	1.300	5.802
	Response Time	0.015	0.012	672.640	518.849
	Mean Ability of Process Level	/	0.454^***^	0.192	0.512
	Ability of Student Level	0.454^**^	/	0.931	0.787
	Correct Responses	0.407^**^	0.985^***^	0.756	0.430

“*31” indicated in “absolute value of difference from 31” in Column 8 refers to the time taken in walking the right route for the item CP007Q02*.

* *p < 0.05*,

** *p < 0.01*,

*** *p < 0.001*.

The result in Table 8 indicates that the sequence of latent classes are consistent with the ability estimates at both process and student levels. For students in the correct group, the mean process-level ability estimate decreased as the number of class shifts, clicks and resets increased. Students with higher process-level ability tended to select the correct route immediately or after a few attempts. Consequently, these students clicked and reset for fewer times because they had a clearer answer in mind and therefore were more certain about it. In contrast, for students in the wrong group, the mean ability estimates at both process and student levels were rather small when the number of class shifts were 0 and 1. When the number of class shifts was 0, students failed to stick with a specific strategy to solve the problem during the process. It took them a longer response time with about two resets on average; as a result, the time cost for their route selection was nearly twice the target time. When the number of class shifts was 1, these students simply stuck to a totally wrong route for the entire time, with shorter response time and fewer numbers of clicks. However, unlike the correct group, the number of class shifts in the wrong group showed a non-linear relationship with the mean ability at both process and student levels. At first, when the number of class shifts increased from 0 to 4, the ability estimates at both levels increased as well. The explanation was that because these students figured out the right routes, they should have higher abilities than the 0 shift group that sticks to the wrong route all the time. For example, students with four shifts all ended up using strategy of Class 1, which was the right strategy class (Appendix 4). Therefore, they were supposed to have the highest process ability in the wrong group. However, when the number of class shifts increased from 5 to 6, the process-level ability estimate dropped. This has much to do with the fact that too many shifts reflected little consideration and a lack of deep cognitive processing.

**Table 8:** Ability estimates and the operational variables in the different numbers of class shifts in the correct group and wrong group.

**Correct or wrong answer group**	**No of class shifts**	**No of students**	**Process-level ability (Mean)**	**Student-level ability (Mean)**	**Response time (Mean)**	**Valid No of click (Mean)**	**Absolute value of difference from 31 (Mean)**	**No of Reset (Mean)**
Correct group (*N* = 307)	1	32	0.650	1.371	714.941	19.375	0	0.156
	2	58	0.692	1.371	609.116	31.655	0	0.121
	3	69	0.468	1.371	814.619	60.667	0	0.275
	4	73	0.196	1.371	601.215	93.795	0	1.192
	5	63	−0.141	1.371	649.711	134.143	0	1.937
	6	12	−−0.279	1.371	679.617	212.25	0	3.5
Wrong group (*N* = 99)	0	11	−0.453	−0.548	991.7	36.909	29.182	2.091
	1	15	−0.439	−0.552	377.713	22.867	1.067	1.067
	2	12	0.139	−0.312	470.392	37.75	1.417	0.5
	3	12	0.466	−0.275	552.042	71.917	0.917	0.667
	4	20	−0.151	−0.438	784.455	94.4	1.250	0.85
	5	24	−0.343	−0.492	690.038	170.292	5.042	2.292
	6	5	−0.348	−0.162	921.02	234	1.000	2.6

## Discussion

A modified MMixIRT model was described for modeling response data at process and student levels. The model developed in this study combined the features of an IRT model, a latent class model, and a multilevel model. The process-level data provide an opportunity to determine whether latent classes or class shifts differ in their response strategies to solve the problem. The student-level data can be used to account for the differences of students' problem solving abilities. The ability estimate at both process and student levels are different across latent classes. The modified MMixIRT model makes it possible to describe differential strategies based on process-level and student-level characteristics. If a student's specific strategies and their strengths and weaknesses can be described in the process of solving a problem, then the assessment of a student's proficiency in problem solving can guide instructional interventions in target areas.

As process data from various computer-based assessment or educational learning system have become common, there is an urgent call for analyzing such data in an accurate way. The psychometrical model-based approach has a great potential in this aspect. Latent classes and the characteristics of latent class shifts obtained from process data can reveal students' reasoning skills in problem-solving. The findings of characteristics of process-level latent classes make it easy to uncover meaningful and interesting action patterns from the process data, and to compare patterns from different students. These findings provide valuable information to psychometricians and test developers, help them better understand what distinguishes successful students from unsuccessful ones, and eventually lead to better test design. In addition, as shown in this study, some operational variables such as the number of resets and the number of clicks or double clicks are related to the ability estimates at both process and student levels and therefore can predict student scores on problem solving assessment. Since students' different abilities capture individual patterns in process data, it can be used to score or validate the rubrics. Williamson et al. (2006) explain that a “key to leveraging the expanded capability to collect and record data from complex assessment tasks is implementing automated scoring algorithms to interpret data of the quantity and complexity that can now be collected” (p. 2).

The extension of the modified MMixIRT approach proposed in this study can be implemented in several ways. Firstly, it can be simplified in removing the process-level ability parameters, and also be extended to include student-level latent classes instead of abilities. Secondly, one of the advantages of this proposed model is that item parameters can be constrained to be equal across the process-level and student-level. So the abilities of both levels are on the same scale and can be compared and evaluated. Lastly, the main benefits of multilevel IRT modeling lie in the possibility of estimating the latent traits (e.g., problem solving) at each level. More measurement errors can be accounted for by considering other relevant predictors such as motivations (Fox and Glas, 2003).

The psychometrical model-based approach also has its limitations. First, even though latent class shifts preserve the sequential information in action series, they do not capture all the related information. For instance, for the purpose of convenient analysis in this study, some unstable characteristics of a latent class such as random shifts were not used in our definition of class characteristics and class shifts. Fortunately, in many cases, as in this study, this missing information does not affect the results. If it becomes an issue in some cases, it can be addressed by considering more details about the latent class shifts to minimize the ambiguity. Second, this study only takes a single route as an analysis unit, yet failing to consider possible route combinations. For example, in some cases two routes are available, it makes full sense to combine these two routes into one to conduct analysis, because the link between these routes is exclusive. In the future, we may consider the transition model for different route combinations, such as Bi-Road. In terms of the generalizability of the modified MMixIRT model for solving complicated problems, if the process data for another single task can be recoded or restructured as the data file in this study, similar models can be applied to explore the latent classes and characteristics of the problem solving process. However, the difficulty during the analysis lies in how to recode the responses into dichotomous data. For multiple tasks, a three-level model can be applied, with the first level as the process level, the second as the task level and the third as the student level. If there are plenty of tasks, the ability estimates of the student will stay stable. Therefore, while the generalizability of the model may be conditional, the main logic of the MMixIRT approach can be generalized.

## Author contributions

HL research design, data analysis, and paper writing. YL paper writing. ML data analysis, and paper writing.

### Conflict of interest statement

The authors declare that the research was conducted in the absence of any commercial or financial relationships that could be construed as a potential conflict of interest.
