# Supplementary material for: Analysis of Process Data of PISA 2012 Computer-Based Problem Solving: Application of the Modified Multilevel Mixture IRT Model
Source: Front Psychol. 2018 Aug 3;9:1372. doi: 10.3389/fpsyg.2018.01372 (PMC6085588; doi:10.3389/fpsyg.2018.01372)
Supplement: Supplementary file 1 [file Data_Sheet_1.pdf]

Appendix1: Example of log data file

cnt	schoolid	StIDStd	event	time	event_number	event_value
ARE	0000189	04832	START_ITEM	797.1000	1.00	NULL
ARE	0000189	04832	click	816.6000	2.00	timeMinutes
ARE	0000189	04832	click	823.1000	3.00	timeMinutes
ARE	0000189	04832	click	824.0000	4.00	timeMinutes
ARE	0000189	04832	click	837.1000	5.00	timeMinutes
ARE	0000189	04832	click	836.2000	6.00	timeMinutes
ARE	0000189	04832	dblclick	836.2000	7.00	timeMinutes
ARE	0000189	04832	click	836.4000	8.00	timeMinutes
ARE	0000189	04832	click	836.9000	9.00	timeMinutes
ARE	0000189	04832	dblclick	836.9000	10.00	timeMinutes
ARE	0000189	04832	ACER_EVENT	843.1000	11.00	'00000000000000000100000
ARE	0000189	04832	click	843.1000	12.00	hit_SakharovNobel
ARE	0000189	04832	ACER_EVENT	846.5000	13.00	'00000000010000000100000
ARE	0000189	04832	click	846.5000	14.00	hit_NobelLee
ARE	0000189	04832	click	851.0000	15.00	map
ARE	0000189	04832	click	852.8000	16.00	map
ARE	0000189	04832	click	856.9000	17.00	map
ARE	0000189	04832	ACER_EVENT	863.9000	18.00	'00001000010000000100000
ARE	0000189	04832	click	863.9000	19.00	hit_LeeMandela
ARE	0000189	04832	ACER_EVENT	865.8000	20.00	'00001000010010000100000
ARE	0000189	04832	click	865.8000	21.00	hit_nowhereSakharov

Appendix2: The number of clicks in each class

	Class1	Class2	Class3	Class4	Class5	Class6
P1	**2107**	**3566**	74	**2128**	43	474
P2	262	582	**2834**	481	**849**	93
P3	0	5	194	0	290	19
P4	12	13	112	16	82	12
P5	**915**	**923**	29	324	9	691
P6	0	2	98	11	275	6
P7	**649**	**536**	173	869	76	687
P8	**1213**	29	21	489	10	346
P9	654	19	**978**	**2285**	40	236
P10	44	**1329**	19	63	10	443
P11	813	367	105	640	526	243
P12	0	6	450	0	358	13
P13	**2138**	**2839**	32	**2174**	32	543
P14	3	552	32	7	622	44
P15	30	19	266	831	89	83
P16	890	12	349	905	378	151
P17	**2216**	35	9	**2296**	26	347
P18	5	**1734**	17	57	8	421
P19	12	254	7	27	445	14
P20	282	11	**1465**	501	25	55
P21	25	572	748	10	**906**	71
P22	31	2	20	39	580	25
P23	2	309	43	25	143	19

Appendix3: The route selection strategy of different classes

	route option strategy
Class1	P1-P13-P17-P8-P5-P7
Class2	P1-P13-P18-P10-P5-P7
Class3	P2-P20-P9-P16-P11;P2-P20-P9-P15-P7
Class4	P1-P13-P17-P9-P16-P11;P1-P13-P17-P9-P15-P7
Class5	P2-P21-P12-P3-P6-P19-P11;P2-P21-P14-P22-P16-P11;
Class6	P7-P5-P10-P18-P13-P1

Appendix4: The sequence of latent classes of students in the correct group

# of process class	# of students	# of student sin different class shfit	Class shift
1	32	32	1[a](32)
2	58	22	21(22)
		3	31(3)
		30	41(30)
		3	61(3)
3	69	16	421(14), 321(2),
		4	231(2), 431(1),531(1)
		45	241(36), 341(9),
		4	261(2), 461(2),
4	73	20	3421(7), 3521(3), 6421(1), 4321(7), 4521(1), 4621(1),
		19	2431(10), 2431(1), 4231(8)
		25	2341(8), 2541(1), 2641(2),3241(7), 3541(1), 6341(1), 5241(2),5341(2),6241(1)
		2	2451(1), 4251(1),
		7	2461(4), 2561(1), 3461(1), 4261(1)
5	63	10	34521(3),34621(1), 45321(2), 53421(2), 35421(1), 43521(1),
		10	24531(1), 54231(2), 42531(2), 42631(1), 52431(3), 26431(1),
		24	23541(4) ,23641(1), 25341(4), 52341(6), 53241(4), 35241(5),
		10	24351(4), 42351(3), 43251(1), 34251(2),
		9	23561(1), 24361(1), 25461(2), 32461(2),34261(2), 54261(1),
6	12	3	624531(1), 562431(1), 635241(1)
		1	562341(1)
		1	364251(1)
		7	352461(1), 435261(3),523461(1),524361(1),534261(1),

Note: The number in bracket presents the number of students who shift from the class; a means class shift during route click, and 1 is for Class 1, 2 for Class 2 and so on. For example, 2461 means that students' action patterns during route click is shifteded from Class 2 to Class 4, and shift to Class 6 from Class 4, and finally switched from Class 6 to Class 1.

Appendix 5: Mplus syntax of the Modified MMixIRT Model

```
VARIABLE:NAMES ARE cnt ID STUID CP1-CP23FP1-FP23;
       USEVARIABLES = CP1-CP23 FP1-FP23;
       CATEGORICAL = CP1-CP23 FP1-FP23;
       BETWEEN = FP1-FP23;
       CLASSES = cb(1) c(6);
       BETWEEN = cb;
       CLUSTER = STUID;
       IDVARIABLE=ID;
DATA:    FILE = data.dat;
ANALYSIS:   TYPE = TWOLEVEL MIXTURE;
       ALGORITHM = INTEGRATION;
       PROCESSORS = 2;
       starts = 200 20;
MODEL:
       %WITHIN%
       %OVERALL%
       fw BY CP1-CP23*;
       %cb#1.c#1%
       fw BY CP1-CP23*;
       [fw@0];
       fw@1;
       %cb#1.c#2%
       fw BY CP1-CP23*;
       [fw];
       fw;
       %cb#1.c#3%
       fw BY CP1-CP23*;
       [fw];
       fw;
         %cb#1.c#4%
       fw BY CP1-CP23*;
       [fw];
       fw;
         %cb#1.c#5%
       fw BY CP1-CP23*;
       [fw];
       fw;
    %cb#1.c#6%
       fw BY CP1-CP23*;
       [fw];
       fw;
       %BETWEEN%
       %OVERALL%
FB BY FP1-FP23*;
       [FB@0];
         FB@1;
       %cb#1.c#1%
       [CP1$1-CP23$1];
       %cb#1.c#2%
       [CP1$1-CP23$1];
       %cb#1.c#3%
       [CP1$1-CP23$1];
```

```
    %cb#1.c#4%
   [CP1$1-CP23$1];
   %cb#1.c#5%
   [CP1$1-CP23$1];
    %cb#1.c#6%
   [CP1$1-CP23$1];
OUTPUT:     TECH1 TECH8;
SAVEDATA: file=FW_FB_C6.dat;
SAVE = fscores;
SAVE = cprobabilities;
```
